# Complex Regional Pain Syndrome Revived by Epileptic Seizure Then Disappeared Soon during Treatment with Regional Intravenous Nerve Blockade: A Case Report

**DOI:** 10.1155/2011/494975

**Published:** 2011-05-03

**Authors:** Masahiko Sumitani, Arito Yozu, Toshiya Tomioka, Satoru Miyauchi, Yoshitsugu Yamada

**Affiliations:** ^1^Department of Anesthesiology and Pain Relief Center, The University of Tokyo Hospital, Tokyo 113-0033, Japan; ^2^Department of Rehabilitation Medicine, Graduate School of Medicine, The University of Tokyo, Tokyo 113-0033, Japan; ^3^Kobe Advanced ICT Research Center, National Institute of Information and Communications Technology, Kobe 651-2492, Japan

## Abstract

We present a case of complex regional pain syndrome (CRPS), in which symptoms, including burning pain and severe allodynia, were alleviated by using a regional intravenous nerve blockade (Bier block) combined with physiotherapy, but reappeared following an epileptic seizure. Symptoms disappeared again following control of epileptic discharges, as revealed by single-photon emission computed tomography (SPECT) and electroencephalography (EEG) results. Although systemic toxicity of a local anesthetic applied by Bier block was suspected as a cause of the first seizure, the patient did not present any other toxic symptoms, and seizures repeatedly occurred after Bier block cessation; the patient was then diagnosed as having temporal symptomatic epilepsy. This case suggests that symptoms of CRPS may be sustained by abnormal brain conditions, and our findings contribute to the understanding of how the central nervous system participates in maintaining pain and allodynia associated with CRPS.

## 1. Introduction

Complex regional pain syndrome (CRPS) causes extreme pain. Dysfunctions of the peripheral nervous system, including the sensory and sympathetic nervous systems, are typically considered to sustain CRPS. The central nervous system (CNS) has also been reported to play an important role in CRPS emergence and maintenance [1]. Although many clinical studies on CRPS and studies using animal models have been conducted, the pathophysiological mechanism of CRPS is not yet clear [2–5]. Here, we report a case of a CRPS patient whose pain was improved by a regional intravenous nerve blockade combined with physiotherapy; however, CRPS relapsed into intolerable pain and severe allodynia following an epileptic seizure. Recurrent CRPS then rapidly improved through the control of epileptic discharges. During epileptic episodes, we investigated the CRPS patient using single-photon emission computed tomography (SPECT) and electroencephalography (EEG). Our findings may contribute to the understanding of how the CNS participates in maintaining CRPS-related pain and allodynia.

## 2. Case Report

A 65-year-old woman with aortic regurgitation following infectious endocarditis had undergone twice aortic valve replacement procedures within 2 months. After the second operation, more than 3 weeks were required before she could be weaned from intensive treatment, including artificial ventilation and sedative drug administration. Following recovery from heart failure, sedative drug administration was discontinued. The patient's clouded consciousness persisted for several days, but she did not show signs of epilepsy. At that time, a computed tomography (CT) scan of the brain showed a diffuse lacunar infarction but no distinct lesion. As the patient's consciousness increased, she complained of intense pain and allodynia originating from the neck and radiating to her left hand. The patient was treated with oral nonsteroidal anti-inflammatory drugs, but no favorable effects were observed. Her pain worsened, and she consulted our pain clinic 3 months after the second operation. She showed no signs and symptoms of neurological complications, except for pain and allodynia, before and after the cardiovascular surgeries.

During the first examination at our pain clinic, she complained of spontaneous burning pain and severe allodynia from her neck to her hand; her left hand was swollen, pale, and hot. We diagnosed her condition as complex regional pain syndrome (CRPS) [6] and began treatment, although the initiating injury was unknown. Because she was being administered anticoagulant therapy, many of the available neural blockades could not be used, except for a regional intravenous nerve blockade (Bier block, with 20 mL of 0.5% lidocaine). This neural blockade was performed 4 times per week concurrently with oral administration of a tricyclic antidepressant, nortriptyline (20 mg); this treatment resulted in the gradual reduction of pain and allodynia, which allowed the patient to receive physiotherapy. One month after treatment, pain and allodynia symptoms were nearly eliminated and swelling had also diminished. Additionally, her upper limb motor disturbance was significantly improved.

**Figure 1:**
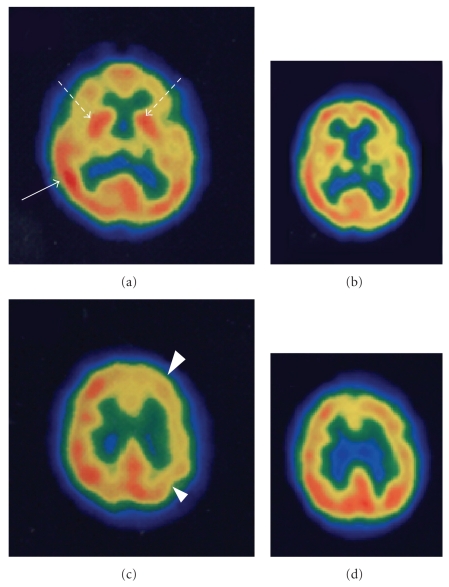
99^m^-HMPAO-SPECT images acquired during a seizure and 10 days later. (a) and (c) show relative hyperperfusion in the right parietal lobe (arrow) and both thalami (dotted arrows) and relative hypoperfusion in the left prefrontal and parietal lobes (arrow heads) during a seizure. (b) and (d) are the same axial slices as (a) and (c), respectively, obtained 10 days after the final seizure. In (b) and (d), relative hyperperfusion and hypoperfusion are not noted, as bilateral symmetrical cerebral perfusion has been normalized.

One hour after the 18th Bier block, the patient suddenly experienced a major epileptic seizure and lost consciousness during physiotherapy. This was the first time in the patient's life that a seizure episode had occurred. Intravenous administration of diazepam followed by thiamylal was used to control the seizure. An emergency CT scan of the brain showed no distinct lesions, which was confirmed by magnetic resonance imaging (MRI). The following day, the patient regained consciousness and complained of burning pain and severe allodynia localized in her left upper limb; subsequently, there was a recurrence of other CRPS symptoms (swelling, skin temperature escalation, and paleness). Despite regular intravenous administration of phenytoin, seizures occurred several times over the following 3 days, which occasionally required additional administrations of intravenous diazepam or thiamylal, and consciousness was again lost. When the patient was partially asleep due to intravenous anticonvulsants, she executed escaping movements for noxious stimulations on various healthy body parts but did not respond to noxious and tactile stimulations on the left upper limb, suggesting an absence of hyperalgesia and allodynia. After 3 days, her consciousness was fully recovered and no seizure episodes occurred. The patient had no further complaints of burning pain or allodynia. SPECT examinations, using 99^m^-Tc-hexamethyl-propylene amine oxime (HMPAO), were performed during the seizure period and 10 days later (Figure 1). The first image, obtained during a seizure, showed relative hyperperfusion in the right parietal lobe and both thalami and relative hypoperfusion in the left frontal and parietal lobes (Figures 1(a) and 1(c)), which improved after 10 days (Figures 1(b) and 1(d)). Electroencephalography (EEG) examinations were also carried out immediately following a seizure and 2 weeks later. EEG readings immediately following a seizure showed irregular and sporadic spiked waves in the left temporal lobe, followed by spikes in both the temporal lobes. Clinically, the patient showed an intermittent left upper limb spasm, although obvious epileptic discharges were not noted on EEG readings after the 3-day seizure episodes. Although postictal EEG readings occasionally displayed irregular spiked waves in the left posterior parietal and temporal lobes, the patient did not show epileptic symptoms. Clinical symptoms suggested that spiked waves were not related to the intermittent left upper limb spasm. On the basis of the SPECT and EEG findings, our neurologist diagnosed the patient as having temporal symptomatic epilepsy focused in the right parietal lobe. Her recovery was uneventful, and pain and allodynia nearly disappeared, although her skin color remained pale and her hand remained hot.

We obtained her consent to report her progress in accordance with the Declaration of Helsinki.

## 3. Discussion

The initiating injury causing CRPS symptoms in our patient was unknown. We speculated that an unconfirmed brachial plexus injury induced by a median sternotomy [7] and/or prolonged immobilization by sedative drug administration [8, 9] may have been a trigger. Furthermore, pain and allodynia may have been derived from disturbed cerebral function, possibly related to the use of heart-lung machines on 2 occasions and for many hours, and infectious endocarditis, which can induce epileptic seizures. Ictal pain related to an epileptic seizure has been noted in approximately 3% of reported epilepsy cases, typically involving an entire limb, a part of a limb, or hemibody [10–12]. Ictal allodynia-related epileptic seizures have been reported in only 2 cases, and both were in children [13, 14].

This is the first known case of successfully treated CRPS revived by an epileptic seizure. Following the first seizure, we suspected systemic toxicity of the local anesthetic (lidocaine, 100 mg) applied by Bier block. However, the anesthetic may not have been responsible for the seizures because the first seizure occurred one hour after Bier block, and the patient did not present symptoms of systemic toxicity to local anesthetics (e.g., change in speech pattern, lightheadedness, dizziness, or agitation) before the seizure, and seizures recurred several times for 3 days following Bier block cessation. The patient's SPECT and EEG abnormal findings were primarily concentrated to the temporal and parietal lobes, whereas epileptic discharges induced by systemic toxicity of local anesthetics are known to generally originate from nonspecific regions of the brain. The patient was therefore diagnosed with temporal symptomatic epilepsy.

A previous report of a brain tumor case suggested that epileptic discharges involved in the main pain pathways (i.e., primary and secondary somatosensory cortices (SI, SII), insula, and amygdale) can cause ictal pain and allodynia [13]. A congenital epilepsy case report suggested that deregulation of pain control established by relative hypoperfusion in the thalamus may play an important role in causing ictal pain and allodynia [14]. Furthermore, acute CRPS is reported to be related to hyperperfusion in the thalamus [15]. On the basis of these findings, we considered that the epileptic discharges noted on the EEG readings and subsequent hyper- and hypoperfusion in the specific brain regions, as revealed by 99^m^-HMPAO SPECT, originated from the right parietal lobe, which includes the two main pain pathway regions (i.e., SI and SII); hyperfusion and hypofusion then spread over the entire brain, including other regions in the pain pathways (i.e., thalamus, anterior cingulate cortex, insula, and amygdala), during the seizure. This abnormal brain condition likely resulted in pain and allodynia. Further, recent advancements in functional brain imaging revealed that the anterior insula, which is strongly associated with autonomic nervous function, is reorganized in CRPS patients [16]. For our patient, abnormal autonomic-like symptoms (i.e., edema, skin discoloration, and skin temperature asymmetry) were revived by epileptic seizures. This may be related to reorganization of autonomic cerebral regions. In the present case, the second episode of CRPS symptoms occurred immediately after the first epileptic seizure episode and then rapidly disappeared with the control of epileptic discharges, although we administered antiepileptic medications, which has little potential to improve neuropathic pain. We therefore concluded that epileptic discharges relapsed into CRPS. Alternatively, we speculate that repeated seizures contributed to improvement of the second bout of CRPS symptoms. For epileptic seizure and pain relief, it has been reported that electroconvulsive therapy (ECT) can be used as an alternative treatment for chronic neuropathic pain [17]. In ECT, epileptic seizures are necessary for pain relief, as fewer seizures are related to a reduced analgesic effect. A possible mechanism of ECT for pain relief may involve alteration of neurotransmitter levels in cerebrospinal fluid, resulting in pain perception modulation. Therefore, we cannot completely rule out the possibility that repeated seizure episodes may have improved the second occurrence of CRPS symptoms.

In conclusion, the present case suggests that a pathophysiological condition(s), such as epileptic discharge and/or abnormal brain perfusion, can repeatedly trigger ictal pain, allodynia, and other signs and symptoms of CRPS when brain regions participating in pain perception have been sensitized. However, why abnormal autonomic-like symptoms of CRPS remain after controlling epileptic discharges and improvement of pain and allodynia is unclear.

## 4. Conclusion

We present a case of CRPS in which the symptoms of burning pain and severe allodynia were once resolved but returned following an epileptic seizure. These symptoms disappeared following the control of epileptic discharges. This suggests that CRPS symptoms may be sustained by abnormal brain conditions, and our results contribute to the understanding of how the CNS participates in maintaining pain and allodynia associated with CRPS.
